# Cyclic AMP binding to a universal stress protein in *Mycobacterium tuberculosis* is essential for viability

**DOI:** 10.1016/j.jbc.2024.107287

**Published:** 2024-04-16

**Authors:** Arka Banerjee, Moubani Chakraborty, Suruchi Sharma, Ruchi Chaturvedi, Avipsa Bose, Priyanka Biswas, Amit Singh, Sandhya S. Visweswariah

**Affiliations:** 1 Department of Developmental Biology and Genetics, Indian Institute of Science, Bengaluru, India; 2 Department of Microbiology and Cell Biology, Centre for Infectious Disease Research, Indian Institute of Science, Bengaluru, India

**Keywords:** cyclic AMP, *Mycobacterium tuberculosis*, *Mycobacterium megmatis*, second messenger, universal stress protein (USP), protein secretion, bacterial signal transduction, Rv1636, MSMEG_3811

## Abstract

Mycobacterial genomes encode multiple adenylyl cyclases and cAMP effector proteins, underscoring the diverse ways these bacteria utilize cAMP. We identified universal stress proteins, Rv1636 and MSMEG_3811 in *Mycobacterium tuberculosis* and *Mycobacterium smegmatis,* respectively, as abundantly expressed, novel cAMP-binding proteins. Rv1636 is secreted *via* the SecA2 secretion system in *M. tuberculosis* but is not directly responsible for the efflux of cAMP from the cell. In slow-growing mycobacteria, intrabacterial concentrations of Rv1636 were equivalent to the concentrations of cAMP present in the cell. In contrast, levels of intrabacterial MSMEG_3811 in *M. smegmatis* were lower than that of cAMP and therefore, overexpression of Rv1636 increased levels of “bound” cAMP. While *msmeg_3811* could be readily deleted from the genome of *M. smegmatis*, we found that the *rv1636* gene is essential for the viability of *M. tuberculosis* and is dependent on the cAMP-binding ability of Rv1636. Therefore, Rv1636 may function to regulate cAMP signaling by direct sequestration of the second messenger. This is the first evidence of a “sponge” for any second messenger in bacterial signaling that would allow mycobacterial cells to regulate the available intrabacterial “free” pool of cAMP.

The genus *Mycobacterium* harbors several diverse species, ranging from free-living members to several pathogenic species, including *Mycobacterium tuberculosis*, the causative agent of human tuberculosis. Tuberculosis alone was responsible for the death of ∼1.6 million people in 2021 (1). It is therefore imperative to understand the physiology of *M. tuberculosis* to identify effective new antimycobacterial strategies.

It has now been well-established that cAMP levels are high in mycobacteria (2, 3), and cAMP is also secreted to the extracellular milieu (2, 3, 4). As seen in many other pathogenic bacteria, *M. tuberculosis* utilizes cyclic AMP-mediated signaling to subvert the host (3, 5, 6), and a remarkably high number of adenylyl cyclases as well as cAMP effector proteins are encoded in its genome (3, 7, 8, 9, 10). However, fast-growing nonpathogenic members, such as *Mycobacterium smegmatis*, also harbor multiple adenylyl cyclases and cAMP-binding proteins (9, 11).

Our earlier work has shown that a significant fraction of intrabacterial cAMP remains protein-bound in both fast and slow-growing mycobacteria (12). Subsequently, we identified specific universal stress proteins (USPs), Rv1636 and MSMEG_3811, in *M. tuberculosis* and *M. smegmatis,* respectively, as abundantly expressed cAMP-binding proteins in these organisms (12). Cyclic AMP binds to cyclic nucleotide-binding (CNB) or GAF domains (13, 14, 15, 16, 17) in proteins. However, the identification of Rv1636/MSMEG_3811 added the USP domain to the list of CNB proteins. USPs are found in bacteria, archaea, fungi, plants, and a few invertebrates, and a subset of them are known to bind ATP (18, 19, 20). Rv1636/MSMEG_3811 formed a new class of USPs that bound cAMP with higher affinity than ATP (12). Interestingly, Rv1636-like cAMP-binding USPs are restricted to *Mycobacterium*, *Corynebacterium,* and *Nocardia* genera.

Rv1636 and MSMEG_3811 show similar biochemical properties, and both bind cAMP with an affinity of 3 μM with a stoichiometry of 1:1 (12). The crystal structure of cAMP-bound MSMEG_3811 revealed how the ATP-binding pocket in the USP-fold can preferentially accommodate cAMP (12). In the present study, we show that Rv1636 is secreted from the cell by the SecA2 pathway and provide evidence that Rv1636/MSMEG_3811 regulates the available pool of cAMP in the mycobacterial cell. Notably, the essentiality of *rv1636* in *M. tuberculosis* is dependent on the ability of this USP to bind cAMP.

## Results

### Genomic organization of *msmeg_3811*/*rv1636* and its expression in mycobacteria

Our earlier report identifying MSMEG_3811 and Rv1636 in *M. smegmatis* and *M. tuberculosis*, respectively, as cAMP-binding proteins (12) added to the repertoire of cAMP-binding proteins in mycobacteria. Additionally, the fact that MSMEG_3811 and Rv1636 bind cAMP through the USP-fold (12) warranted further investigation into the cellular functions of these USPs in mycobacterial physiology.

**Figure 1:**
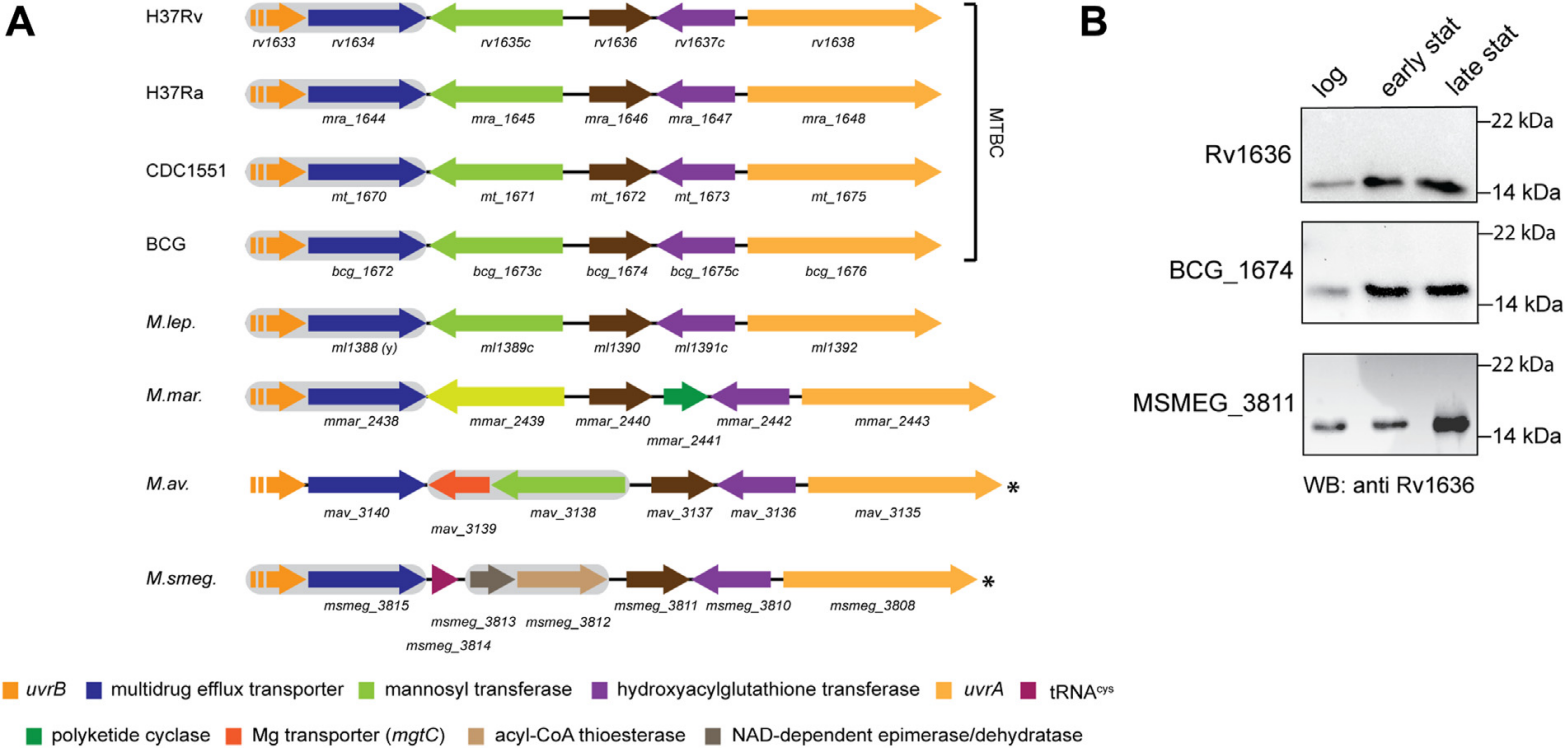
**Genomic organization and expression of *rv1636* and its orthologs.***A*, schematic overview of the locus encoding *rv1636* and its mycobacterial orthologs. Orthologs are similarly color-coded with *arrowheads* indicating gene orientations. Genes predicted to occur in operons are highlighted in *gray*. A *discontinuous arrow* indicates a partial representation of the gene. (ψ) indicates pseudogene, and *asterisks* indicate the genomic orientation in the negative strand. Gene lengths are not to scale. MTBC, *Mycobacterium tuberculosis* complex; H37Rv, *M. tuberculosis* H37Rv; H37Ra, *M. tuberculosis* H37Ra; CDC1551, *M. tuberculosis* CDC1551; BCG, *M. bovis* BCG; *M.lep*., *M. leprae* TN; *M.mar*., *M. marinum*; *M.av*., *M. avium*; *M.smeg*., *Mycobacterium smegmatis* mc^2^ 155. *B*, expression of Rv1636, BCG_1674, and MSMEG_3811 as a function of growth. Immunoblot was performed using antisera raised against recombinant Rv1636. Log, logarithmic; stat, stationary. The data shown are representative of experiments performed thrice.

In all members of the *M. tuberculosis* complex (MTBC), *rv1636,* and its orthologs are encoded as a monocistron, with the identical organization of flanking genes in this locus (Fig. 1*A*). Interestingly, in *Mycobacterium leprae*, the *rv1636* ortholog, *ml1390*, is predicted to be a functional gene with conservation of the genomic locus (Fig. 1*A*). Further, in all mycobacterial species, *rv1636* orthologs are orientated in the direction of the movement of the replication fork, accounting in part for high expression levels in mycobacteria. Additionally, in a recent bioinformatic analysis, it was revealed that head-on genes include antibiotic resistance and virulence genes and are often under positive selection (21).

We monitored the expression of Rv1636 and its orthologs from two mycobacterial species, *Mycobacterium bovis* Bacillus Calmette Guérin (BCG) and *M. smegmatis*. Rv1636 levels showed a marginal increase as the cells entered the stationary phase and were maintained even in the late stationary phase (Fig. 1*B*). Interestingly, the gene sequences of *rv1636* and *bcg_1674* and adjacent 1000 bp upstream and downstream are identical. It is noteworthy that USPs from diverse bacterial species also show higher protein expression levels in the stationary phase of growth (22, 23).

### Secretion of Rv1636 is SecA2-dependent

Before we identified Rv1636 as a cAMP-binding protein, several independent proteomics-based studies had reported Rv1636 to be a secreted protein both *in vitro* and in animal models of infection, including sera from human smear-positive tuberculosis patients (24, 25, 26, 27, 28, 29). Studies with *M. bovis* BCG also showed BCG_1674 to be secreted (25, 27). However, the mechanism of secretion of Rv1636 (and its orthologs) remained unclear. Rv1636 lacks any canonical signal peptide at its N terminus, ruling out secretion *via* the SecA1 and Tat pathways. Rv1636 is not located in any of the ESAT-6 secretion system (ESX) loci, and since *M. bovis* BCG, a strain inherently lacking the ESX-1 system also secretes BCG_1674 (Fig. 1*B*), secretion by the ESX-1 system is ruled out.

**Figure 2:**
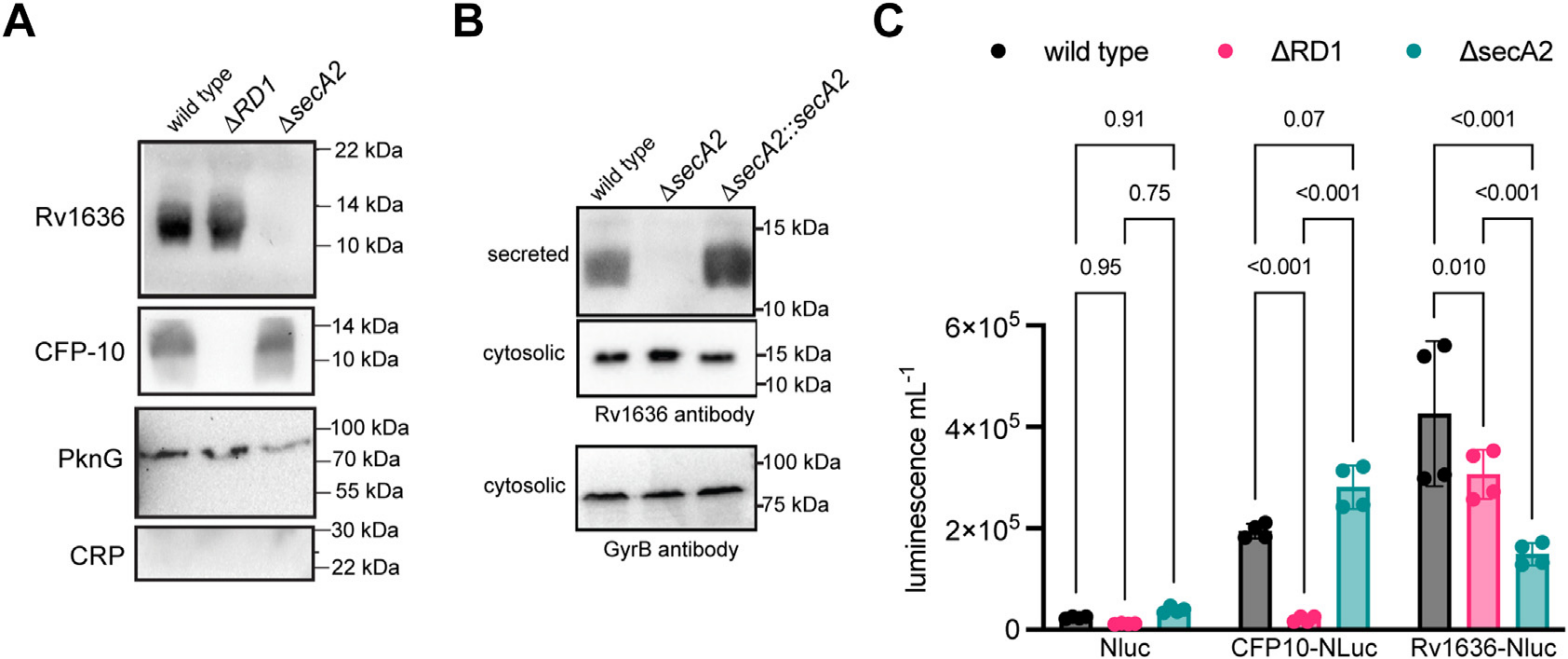
**Secretion of Rv1636 is SecA2-dependent.***A*, immunoblots probing for Rv1636, CFP-10, PknG, and CRP in fractions prepared from cultures of *Mycobacterium tuberculosis* WT, *ΔRD1,* and *ΔsecA2* strains. *B*, Rv1636 levels were measured in the culture filtrate (secreted), cell envelope (for PknG) and cytosol as detected by immunoblotting in *M. tuberculosis* WT, *ΔsecA2* and *ΔsecA2::secA2* complemented strains, respectively. Cytosolic protein loading was normalized to gyrase B levels. Data shown in (*A* and *B*) are representative blots of experiments performed thrice. *C*, secretion of Nluc, CFP10-NLuc, and Rv1636-Nluc proteins was monitored where *nluc* and *rv1636-nluc* were driven by the *rv1636* promoter and *cfp10-nluc* was driven by the *cfp10* promoter. Mean ± SD is plotted for duplicate cultures across two biological replicates. Two-way ANOVA with Tukey’s multiple comparison test was performed, and *p* values are shown. CRP, cAMP receptor protein.

Reported substrates of the SecA2 pathway in *M. tuberculosis*, *viz.* SodA and KatG lack a canonical signal peptide (30, 31). We, therefore, asked whether Rv1636 secretion was SecA2-dependent. We monitored the presence of Rv1636 in culture filtrates prepared from *M. tuberculosis* WT, *ΔsecA2,* and *ΔRD1* strains using anti-Rv1636 antisera (Fig. 2*A*). Rv1636 was seen in the fractions prepared from WT and *ΔRD1* strains but was absent from the culture filtrate fraction of the *ΔsecA2* strain (Fig. 2*A*). As a control, CFP-10 could be detected in the culture filtrate prepared from the *ΔsecA2* strain since secretion of CFP-10 is dependent on proteins encoded by the *RD1* locus (32). Further, PknG, a SecA2 substrate (33), was detected in the cell envelope fraction of the *ΔRD1* strain but levels were markedly decreased in fractions prepared from the *ΔsecA2* strain. Finally, cAMP receptor protein (CRP) antibodies were used to confirm that the protein in the culture filtrate was not a consequence of cell lysis since CRP is exclusively an intrabacterial protein (Fig. 2*A*). Therefore, we propose that Rv1636 is secreted *via* the SecA2 secretion pathway.

Complementation of SecA2 in the *ΔsecA2* strain restored Rv1636 expression in the culture filtrate (Fig. 2*B*), confirming that secretion of Rv1636 was mediated by SecA2. We also observed that the cytosolic levels of Rv1636 appeared to be higher in the *ΔsecA2* strain than in WT and the complemented strains (Fig. 2*B*), suggesting intrabacterial accumulation of the cytosolic variant in the absence of secretion.

We noted that secreted Rv1636 migrated faster than its cytosolic counterpart (Fig. 2*B*). The first six amino acids of secreted Rv1636 are missing, as revealed by N-terminal sequencing (25). Therefore, secretion-dependent protein cleavage of Rv1636 may occur since no evidence of the shorter form was seen in the cytosol.

We developed a novel nanoluciferase (NanoLuc/Nluc)-based assay to monitor protein secretion in mycobacteria (34). To validate this assay, we fused CFP-10 at its C terminus with nanoluciferase (CFP-10-Nluc) with an intervening flexible linker of sequence GGSGGGGSGGGSSGG (34). The C terminus of CFP-10 in complex with ESAT-6 is long and flexible (35) and the last seven amino acids are important for interaction with Rv3871, a cytosolic component of the ESX-1 system (36). We argued that the presence of the linker sequence would retain the flexibility of the C terminus of CFP-10 and allow critical, if not optimal interactions, with the secretion machinery. The CFP-10-Nluc construct and a control plasmid in which the *rv1636* promoter drove the expression of nanoluciferase alone were introduced into WT, *ΔRD1,* and *ΔsecA2* strains. Low luciferase activity was detected in the strains harboring the nanoluciferase gene driven by the Rv1636 promoter, showing that nanoluciferase was not efficiently secreted from mycobacterial cells (Fig. 2*C*). However, luciferase activity was detected in the supernatant of the WT strain harboring CFP10-Nluc, while a marked reduction in luciferase activity was seen in supernatants prepared from the *ΔRD1* strain (Fig. 2*C*). In contrast, no reduction in luciferase activity was seen in the culture supernatant when CFP10-Nluc was expressed in the *ΔsecA2* strain, indicating that the secretion of CFP10-Nluc was dependent on the RD1 locus. This established that our assay could effectively reflect protein secretion in mycobacterial cells.

We then tested secretion of Rv1636 using an Rv1636-Nluc construct. (Fig. 2*C*). High luciferase activity was detected in the culture supernatant of the WT strain harboring Rv1636 fused to nanoluciferase (Rv1636-Nluc). A slight but significant reduction was seen in luciferase activity in the *ΔRD1* strain, but a more than 50% reduction was seen in the *ΔsecA2* strain. This confirmed that secretion of Rv1636 is largely SecA2-dependent.

### Intrabacterial, bound cAMP is directly correlated with levels of Rv1636/MSMEG_3811

**Figure 3:**
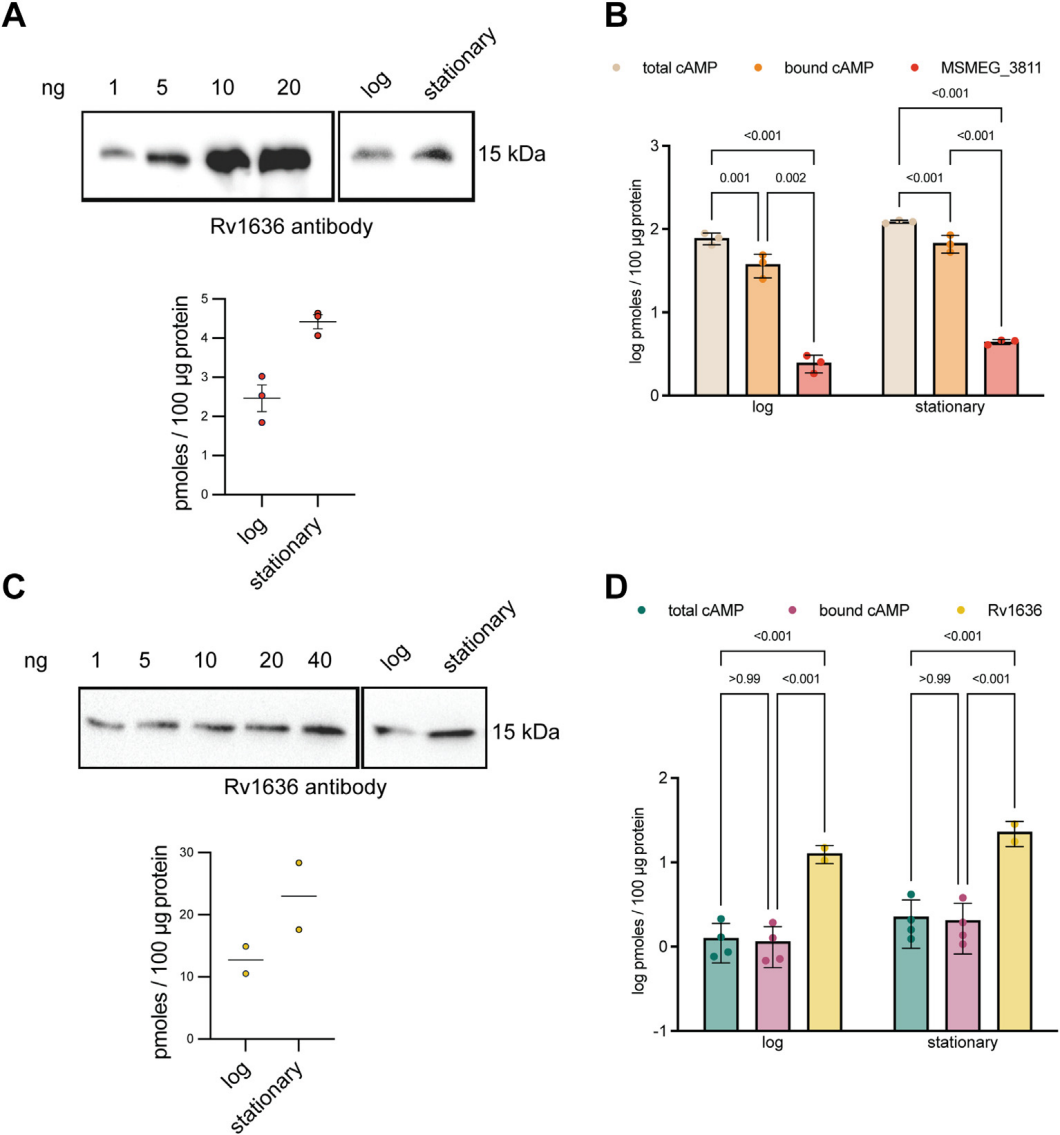
**Determination of cytosolic levels of MSMEG_3811, Rv1636, and intrabacterial cAMP.***A*, Western blot with varying amounts of MSMEG_3811 loaded from which a standard curve was generated following densitometric scanning. *Lower panel*: blots with lysates prepared from the log and stationary phase of growth of *Mycobacterium smegmatis* were used to estimate the concentration of MSMEG_3811 by interpolation from the standard curve. The data shown are representative of experiments performed thrice and values from individual experiments are shown ± SD. *B*, levels of total and bound cAMP in lysates used for Western blotting were estimated and compared to the level of MSMEG_3811 at different phases of growth. The data shown are from experiments performed at least twice in duplicate. *C*, Western blot with varying amounts of Rv1636 loaded from which a standard curve was generated following densitometric scanning. *Lower Panel*: blots with lysates prepared from the log and stationary phase of *Mycobacterium tuberculosis* growth were used to estimate the concentration of Rv1636 by interpolating from the standard curve. The data shown are representative of experiments performed twice, and values shown are the mean ± SD. *D*, levels of total and bound cAMP in lysates prepared from *M. tuberculosis* and used for Western blotting were estimated and compared to the level of Rv1636 at different phases of growth. The data shown are from experiments performed at least twice in duplicate. Rv1636 levels were higher than that of intrabacterial cAMP.

To investigate whether levels of Rv1636/MSMEG_3811 were sufficient to bind the available cAMP in the mycobacterial cell, the amount of intrabacterial MSMEG_3811 in WT *M. smegmatis* cells was estimated using known amounts of recombinant MSMEG_3811 and densitometric analysis of bands seen on Western blotting (Fig. 3*A*). In logarithmic and stationary growth phases, the absolute levels of MSMEG_3811 were estimated as 1 to 3 pmole per 100 μg of total protein, respectively (Fig. 3*A*). However, the total and bound cAMP levels in *M. smegmatis* were significantly higher and averaged around 50 to 80 pmol/100 μg protein (Fig. 3*B*). This suggested that in *M. smegmatis*, cAMP could be bound to proteins other than MSMEG_3811, thereby contributing to the bound cAMP fraction.

In slow-growing pathogenic *M. tuberculosis*, levels of intrabacterial Rv1636 ranged from ∼10 to 20 pmol per 100 μg of total protein at logarithmic and stationary growth phases (Fig. 3*C* and *D*). Interestingly, cAMP levels in *M. tuberculosis* appeared much lower, ranging between ∼1 and 2 pmol per 100 μg of protein (Fig. 3*D*). Thus, almost the entire fraction of intrabacterial cAMP in *M. tuberculosis* was protein-bound (Fig. 3*D*) similar to the findings we have reported earlier in *M. bovis* BCG (12). This suggests that Rv1636 could be the significant cAMP-binding protein in this species, and the amount of free cAMP available to other cAMP effectors in *M. tuberculosis* may be tightly regulated by levels of Rv1636 in the cell.

### Rv1636/MSMEG_3811: An intrabacterial sponge for cAMP

Cyclic AMP-binding to the GAF or CNB domains in proteins relays the signal to an associated effector domain that carries out downstream functions in a signaling cascade (14, 15, 16). In contrast, in Rv1636/MSMEG_3811, the USP domain to which cAMP binds is not associated with any other functional or catalytic domain. We, therefore, hypothesized that Rv1636/MSMEG_3811 may act to sequester the cyclic nucleotide.

In contrast to slow-growing *M. bovis* BCG (12) and *M. tuberculosis*, where almost the entire cytosolic cAMP was protein bound (Fig. 3*D*; 1.27 ± 0.62 pmol/100 μg protein total cAMP *versus* 1.15 ± 0.59 pmol/100 μg protein bound cAMP; *p*-value > 0.99), fast-growing *M. smegmatis* could be used to test our hypothesis, since ∼50% of the intrabacterial cAMP in this species exists as a bound fraction (Fig. 3*B*; 77.3 ± 12.4 pmol/100 μg protein total cAMP *versus* 37.9 ± 11.9 pmol/100 μg protein bound cAMP; *p*-value 0.001). Therefore, overexpression of MSMEG_3811/Rv1636 in cells should sequester more intrabacterial cAMP.

**Figure 4:**
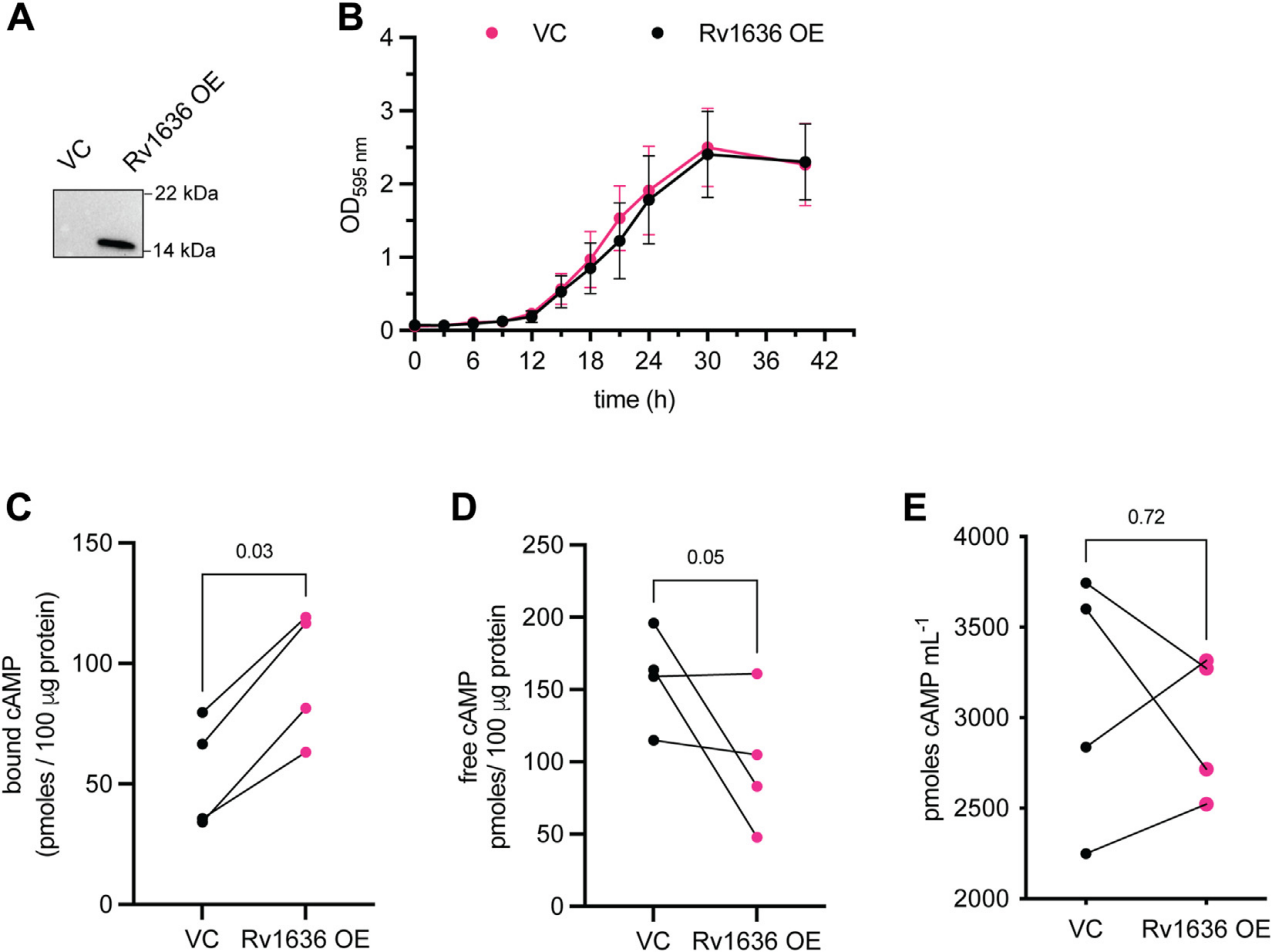
**Overexpression of Rv1636 increases bound intrabacterial cAMP levels in *Mycobacterium smegmatis*.***A*, immunoblot showing overexpression of Rv1636 in WT *M. smegmatis* mc^2^ 155. Blot shows lysates prepared from *M. smegmatis* cells transformed with either a vector control (VC) or Rv1636 overexpressing plasmid (OE) and exposed to show the extent of overexpression. The blot shown is representative of two independently grown colonies. *B*, growth curves of Rv1636 OE and VC strains. Cultures in duplicate were grown for the indicated time, and aliquots were taken for measuring absorbance at 595 nm. Mean ± SD is plotted for experiments performed twice. *C*, bound cAMP in the intrabacterial fractions of Rv1636 OE and VC strains. Cultures were grown in duplicate, and individual data for experiments performed twice are shown. Data were analyzed using a one-tailed *t* test with Welch’s correction (*D*). Free cAMP levels measured in the cytosol from the same cultures used in (*C*). Data were analyzed using a one-tailed *t* test with Welch’s correction. *E*, extracellular cAMP levels in the strains used for measuring bound and free cAMP. Data were analyzed using a two-tailed *t* test with Welch’s correction.

Overexpression of Rv1636 under its promoter from a low-copy number episomal plasmid (Fig. 4*A*) revealed no difference in the growth of the cells with respect to the vector control (Fig. 4*B*). We separated the intrabacterial cAMP into protein-bound and free fractions as described earlier (37) to monitor levels of cAMP. There was a significant increase in bound cAMP levels following overexpression of Rv1636 across four experiments (95 ± 27 pmol cAMP/100 μg protein in the overexpressing strain *versus* 54 ± 23 pmol cAMP/100 μg protein in the vector control strain; Fig. 4*C*). Concomitantly, there was a reduction in “free” cAMP in the strains overexpressing Rv1636 (99 ± 47 pmol cAMP/100 μg protein *versus* 158 ± 33 pmol cAMP/100 μg protein; Fig. 4*D*). Levels of extracellular cAMP are very high in cultures of *M. smegmatis* (2). No significant difference in secreted cAMP was observed in the strains overexpressing Rv1636 (2957 ± 398 pmol cAMP/ml in the overexpressing strain *versus* 3108 pmol cAMP/ml in the vector control strain; Fig. 4*E*). Thus, levels of Rv1636 in the cell determine the levels of “free” cAMP.

**Figure 5:**
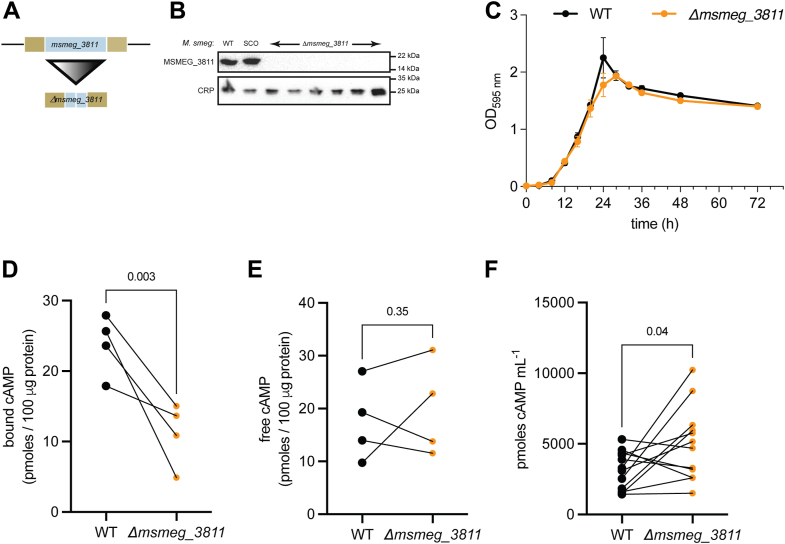
**Deletion of *msmeg_3811* leads to reduced bound intrabacterial cAMP levels.***A*, schematic depiction of the WT *msmeg_3811* (*top*) and *Δmsmeg_3811* (*bottom*) alleles. The *blue bar* represents the *msmeg_3811* ORF in both the WT and *Δmsmeg_3811* strains. The *brown bars* represent the flanking sequences used for the homologous recombination. Image not to scale. *B*, immunoblot confirming the *Δmsmeg_3811* strains by probing with anti-Rv1636 antisera. WT and SCO strains were used as a positive control. Normalization for protein loading was performed using CRP antibodies. *Mycobacterium smegmatis* strains. SCO, single crossover. The data shown are representative of blots performed twice. *C*, growth of WT and *Δmsmeg_3811* strains. Mean ± SEM for two biological duplicates plotted from a representative experiment performed twice (*D*) bound cAMP in the intrabacterial fractions of WT and *Δmsmeg_3811* strains. Cultures were grown in duplicate, and individual data for experiments performed twice are shown. Data were analyzed using a one-tailed *t* test with Welch’s correction (*E*). Free cAMP levels were measured in the cytosol from the same cultures used in (*D*). Data were analyzed using a one-tailed *t* test with Welch’s correction. *F*, extracellular cAMP levels in the strains used for measuring bound and free cAMP. Data were analyzed using a two-tailed *t* test with Welch’s correction. CRP, cAMP receptor protein.

We then asked if deletion of *msmeg_3811* gene would result in a decrease in bound cAMP. We generated an unmarked deletion strain of *msmeg_3811* (*Δmsmeg_3811*) (Fig. 5, *A* and *B*). Deletion of *msmeg_3811* did not affect the growth (Fig. 5*C*) of *M. smegmatis* under the conditions of growth used in our experiments. However, the intrabacterial bound cAMP levels in *Δmsmeg_3811* were found to be significantly lower than the WT cells (11 ± 4.5 pmol cAMP/100 μg protein *versus* 24 ± 4.3 pmol cAMP/100 μg protein in WT cells; Fig. 5*D*). Levels of free cAMP in the *Δmsmeg_3811* were not changed significantly (20 ± 9 pmol cAMP/100 μg protein in the *Δmsmeg_3811* strain *versus* 18 ± 7.5 pmol cAMP/100 μg protein in the WT strain; Fig. 5*E*), but a marginal increase in total extracellular cAMP levels (5002 ± 2605 pmol cAMP/ml in the *Δmsmeg_3811* strain *versus* 3134 ± 9 pmol cAMP/ml in the WT strain (Fig. 5*F*) was seen. Taken together, overexpression of Rv1636 and deletion of *msmeg_3811* provide support for our hypothesis that Rv1636/MSMEG_3811 may sequester cAMP, thus regulating or tuning free cAMP concentrations in the cells and the amount secreted by mycobacteria.

### *rv1636* is an essential gene for survival of *M. tuberculosis*

We have seen that almost all cAMP in slow-growing mycobacteria remained bound to protein. Concentrations of Rv1636 were high enough to serve as the cAMP sponge in these bacteria (Fig. 3*D*). While the deletion of MSMEG_3811 would have only marginally increased free cAMP in the cell, the consequences resulting from deletion of *rv1636* may be more profound if Rv1636 was responsible for maintaining “bound” levels of cAMP. We, therefore, attempted to generate an unmarked deletion of *rv1636* in *M. tuberculosis* using a two-step, homologous recombination-based strategy (38). We confirmed two independent single crossover (SCO) colonies for the *rv1636* locus in WT *M. tuberculosis* by PCR and Southern blotting (Fig. S1). Once the SCOs were confirmed, they were grown for the second crossover to occur, followed by subsequent selection of the second crossovers (38). Since the two crossover events are selected in a stepwise manner independent of each other, the expected outcome after the second crossover is to get both WT and KO colonies in a 1:1 ratio (38). Surprisingly, all the 47 legitimate double crossover (DCO) colonies screened appeared to be WT, suggesting that *rv1636* may be an essential gene.

We generated a merodiploid strain (*Mtb::rv1636*) where we introduced a second functional, WT copy of *rv1636* at the mycobacteriophage L5 *att* site under its promoter (Fig. S1, *A* and *B*). Rv1636 levels were detected by immunoblotting (Fig. S1*C*). We then deleted the copy of *rv1636* at the endogenous locus in the merodiploid strain. We could readily generate a deletion of *rv1636* at the endogenous site (Fig. S1*D*) while the L5 *att* site copy remained intact (Fig. S1*D*). This strongly suggested that under the conditions of growth we were using, *rv1636* is an essential gene.

**Figure 6:**
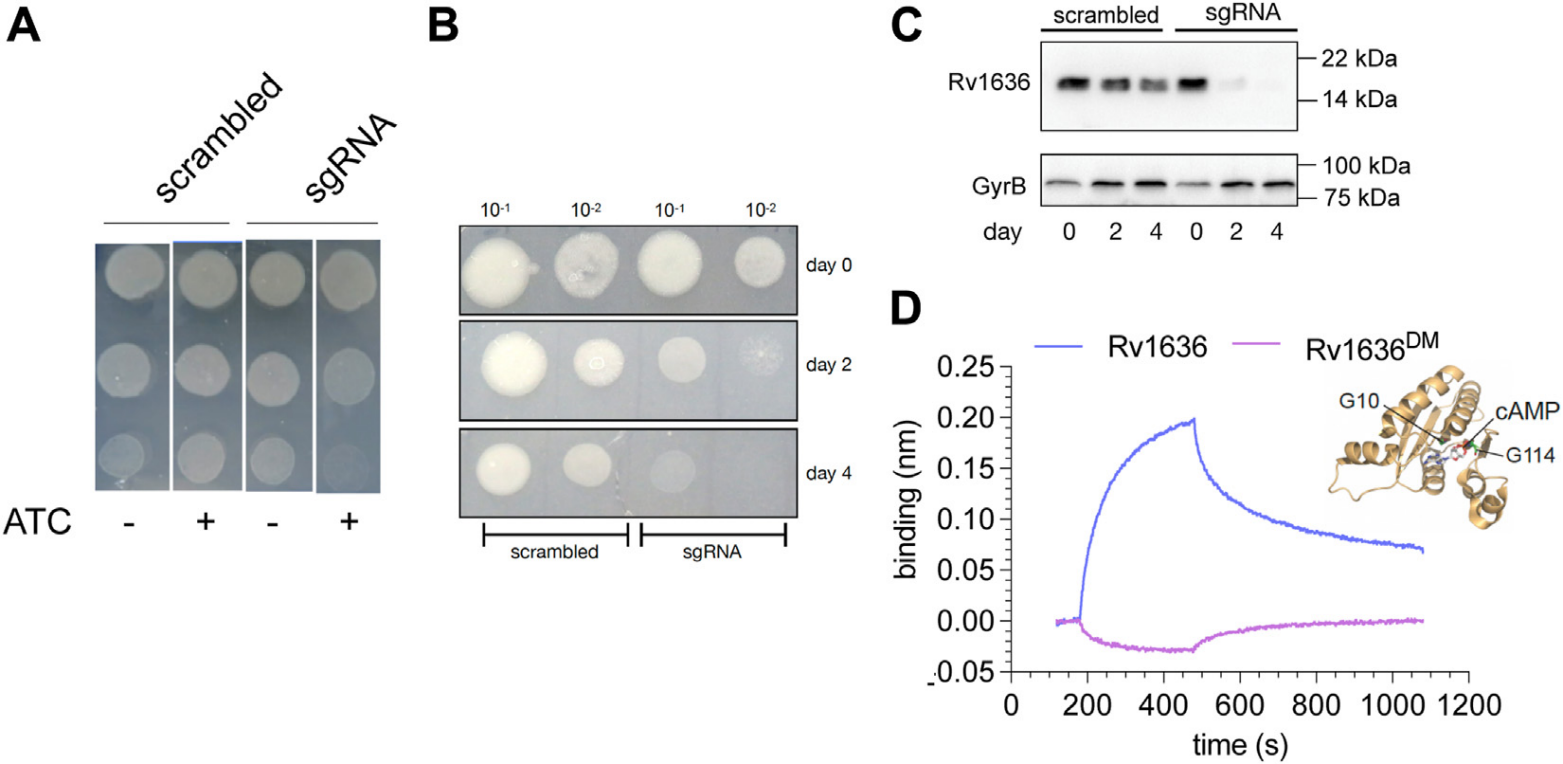
**With one supplement.Essentiality of rv1636 in *Mycobacterium tuberculosis****.* (*A*) CRISPRi (Spy dCas9)-mediated knockdown of rv1636 using a rv1636-targeting sgRNA, which binds spanning the +11 to +30 position within the rv1636 ORF. Upon induction of CRISPRi in the presence of ATC, severe growth retardation was observed only with the specific sgRNA targeting rv1636. The data shown are representative of experiments performed thrice. *B*, spot assay corresponding to the immunoblot shown in (*C*). *C*, Rv1636 protein levels in cells with rv1636-specific sgRNA or scrambled control. The blot is representative of lysates from a single experiment, with the experimental knockdown performed thrice. Protein in each lane was monitored by a blot probed with gyrase B antibody. *D*, cyclic AMP binding of Rv1636 and Rv1636 G10TG113A (Rv1636^DM^) was measured using biolayer interferometry. The data shown are representative of experiments performed twice with individual protein preparations. The inset shows the crystal structure of a cAMP-bound MSMEG_3811 monomer (from PDB ID 5AHW), indicating the positioning of G10 and G114 (the equivalent of G113 in Rv1636) residues in the cAMP-binding pocket. ATC, anhydrotetracycline; PDB, Protein Data Bank; sgRNA, single-guide RNA.

To confirm this, we designed a CRISPRi-mediated knockdown of *rv1636* in WT *M. tuberculosis* cells (39). The single-guide RNA (sgRNA) specific to *rv1636* could anneal from the +11 position in the ORF, halting gene transcription. When the *rv1636*-specific sgRNA was induced with anhydrotetracycline (ATC), the cells showed severe growth retardation as opposed to scrambled sgRNA (Fig. 6*A*). We monitored the expression of Rv1636 by Western blotting with lysates harvested on days 2 and 4 after ATC addition and observed almost complete depletion of Rv1636 within 48 h (Fig. 6, *B* and *C*). Together, these results indicate that the *rv1636* gene was essential for the survival of *M. tuberculosis*.

Next, we asked whether the essentiality of *rv1636* was linked to its ability to bind cAMP. The crystal structure of the cAMP-bound MSMEG_3811 (Protein Data Bank [PDB] ID 5AHW) aided in identifying residues that made direct contact with the ligand (12). Gly-10 and Gly-114 make interactions with the 2′-OH of the ribose moiety and the cyclic phosphodiester, respectively (Fig. 6*D* inset), and both the corresponding single mutants in Rv1636 - G10T and G113A—showed reduced cAMP binding (12). We mutated both residues for this study to generate a double mutant (Rv1636^DM^) variant. The recombinant Rv1636^DM^ protein lost all cAMP binding, as monitored using biolayer interferometry (BLI) (Fig. 6*D*).

We generated a merodiploid strain (*Mtb::rv1636*^*DM*^) where we provided the *rv1636*^*DM*^ allele as a second copy at the mycobacteriophage L5 *att* site. The chromosomal integrity of the merodiploid strain was confirmed by PCR and Southern blotting (Fig. S1, *A* and *B*) and the expression of Rv1636^DM^ was quantitated by immunoblotting (Fig. S1*C*). We tested whether deletion of the endogenous *rv1636* allele was possible in the background of the *rv1636*^*DM*^. Of a total of 23 legitimate DCO colonies screened, no KO was obtained. Therefore, this genetic evidence allows us to conclude that the essentiality of *rv1636* was dependent on its ability to bind cAMP.

## Discussion

Mycobacterial genomes encode a complex cAMP synthesizing, utilizing, and degrading machinery (3, 9, 40), emphasizing the diversity in how this ancient small molecule is deployed in regulating bacterial physiology. For example, changes in cAMP levels affect the utilization of long-chain fatty acids, an important carbon source for intracellular mycobacteria (10). Further, an increase in cAMP levels decreases cholesterol utilization (41). The exact mechanisms by which cAMP regulates these effects presumably requires the regulated activities of cAMP-binding proteins. The CNB domain in these proteins are associated with effector domains that have acyltransferase activity, DNA binding or predicted ATPase activities (3). Many remain uncharacterized and the coordinated regulation of their functions to bring about a phenotypic change remains to be investigated.

However, the activity of these cAMP binding effectors depends on the availability of “free” cAMP, which is not bound to any other protein. The addition of Rv1636/MSMEG_3811, a single-domain protein that binds cAMP *via* the USP-fold, is unique and intriguing. The absence of any associated domain together with the fact that Rv1636/MSMEG_3811 did not copurify with any other major protein on cAMP-affinity chromatography (12), suggested that the role of Rv1636/MSMEG_3811 was to sequester cAMP in the cells. Both overexpression of Rv1636 and deletion of *msmeg_3811* in *M. smegmatis* supported this hypothesis. The mycobacterial replicon in the episomal plasmid used to overexpress Rv1636 typically has a copy number of ∼5 in mycobacterial cells (42, 43). Given the similarities in biochemical properties of Rv1636 and MSMEG_3811 (12), an increase of ∼40 to 45 pmol of bound cAMP/100 μg of protein following the overexpression of Rv1636, and the reduction of ∼10 pmol of bound cAMP/100 μg of protein in the *Δmsmeg_3811* cells are in agreement with the hypothesis.

The observation that neither overexpression of Rv1636 nor deletion of *msmeg_3811* had any effect on the growth of the *M. smegmatis* cells can be explained by the fact that the total, as well as free cAMP levels in fast-growing *M. smegmatis*, are far higher than the levels of MSMEG_3811 protein in the cell. In contrast, in *M. tuberculosis*, both intrabacterial cAMP and Rv1636 concentrations were comparable. Thus, removal of Rv1636 from the cell would release cAMP and make it available for other intracellular effectors. An earlier report by Schubert *et al.* (44) demonstrated that absolute levels of Rv1636 appeared to be 6.2 pmol per 100 μg of protein, making it the 20th most abundant protein in the cell. Genetic analyses revealed that *rv1636* is essential for the survival of *M. tuberculosis,* and the ability of Rv1636 to bind cAMP was critical to allow the viability of the cells. Thus, Rv1636 may function upstream of other canonical cAMP-binding proteins in *M. tuberculosis* by regulating the cAMP available for binding to other cAMP-binding effectors.

Results from transposon insertion mutagenesis screens (45, 46) predicted that *rv1636* was an essential gene. Of the ten permissive TA insertion sites found within the *rv1636* gene, only one insertion was observed after the TA of the stop codon in the ORF, leaving the entire ORF unperturbed (45, 46). In the promoter and 5′-UTR of *rv1636*, TA sites are located at −3, −25, −43, −58, −60, −64, and −74 positions, respectively. Except for the −3 site, all sites had transposon insertions (45, 46) indicating that transcription could start from the +5 position (47). The study by Griffin *et al.* (46) utilized minimal media plus glycerol. In contrast, DeJesus *et al.* (45) had grown *M. tuberculosis* on 7H9 or 7H10 media containing oleic acid-albumin-dextrose-catalase (OADC) supplement, suggesting that the essentiality of *rv1636* may be independent of the growth media used or the culture conditions. This remains to be investigated in the future.

A total of six of the ten USPs in *M. tuberculosis* fall under the DosR-regulon (48), and Rv1636 is not one of those, suggesting its unique functional relevance. The presence of the +5 transcription start site identifies *rv1636* as one of the 47 genes which can be transcribed as both leadered and leaderless transcripts (47). The smaller secreted form of Rv1636 may be the translated product of the leaderless transcript or have undergone proteolytic cleavage during secretion. SodA, KatG, and components of the Mce transporter are known SecA2 substrates in *M. tuberculosis* (31, 49, 50) to which we now add Rv1636. In *Mycobacterium marinum* and *M. tuberculosis*, secretion of PknG is SecA2-dependent (33, 51). The role of secreted Rv1636, however, remains to be elucidated. The fact that *secA2* mutant exhibits attenuated virulence (30, 31, 50, 52) warrants further investigation into the functions of secreted Rv1636.

None of the other cAMP-binding protein-encoding genes or other *usp* genes are predicted to be essential in *M. tuberculosis* (45, 46). The CRP/*rv3676* gene is attenuated in its virulence (53), while the KO of *kat*/*rv0998 in M. bovis* BCG shows a growth defect when grown in propionate as the sole carbon source (54). Individual deletions of 4 *usp* genes – *rv1996*, *rv2005c*, *rv2026c,* and *rv2028c*, respectively, in *M. tuberculosis* H37Rv showed no growth disadvantage (55). Deletion of another *usp*, *rv2623*, in *M. tuberculosis* Erdman strain failed to establish chronic infection in guinea pig and mice models, showing a hypervirulence phenotype (56). This was linked to the ability of Rv2623 to be able to bind ATP (56). The ortholog of *rv1636* in *M. leprae*, *ml1390*, is the only functional *usp* gene encoded in its genome (55).

Approximately 600 to 700 genes in *M. tuberculosis* genome are predicted to be essential for the survival of the bacteria, accounting for ∼16% of all genes (45, 46, 57). The inclusion of *rv1636* in this list suggests that Rv1636 may be a potent target for developing antimycobacterial drugs, given the complete absence of USPs in higher mammals. Our docking results with a natural compound that binds Rv1636 (58) suggest that targeting Rv1636 may prove to help design novel therapeutics for the treatment of tuberculosis.

## Experimental procedures

### Strains and culture conditions

*M. smegmatis* mc^2^ 155 cells were grown in Middlebrook 7H9 medium (BD Biosciences) supplemented with 0.2% glycerol and 0.05% Tween 80 at 37 °C with shaking at 200 rpm. *M. bovis* BCG and *M. tuberculosis* H37Rv cultures were grown in the same medium containing OADC supplement at a final concentration of 10% (v/v) in tissue culture flasks at 37 °C in a humidified incubator in bio-safety level 2 (BSL2) and bio-safety level 3 (BSL3) facilities, respectively. For solid media, *M. smegmatis* cells were grown in Middlebrook 7H10 agar (BD Biosciences) containing 0.5% glycerol, and *M. tuberculosis* cells were grown in Middlebrook 7H10 agar supplemented with 0.5% glycerol and 10% OADC. As and when required, kanamycin and hygromycin were used at 20 and 50 μg/ml concentrations, respectively.

PCR was carried out to generate the SecA2 complemented strain using genomic DNA of *M. tuberculosis* H37Rv. The *secA2* gene was amplified in two parts, and PCR amplicons ligated into cloning vector pCR2.1 (Thermo Fisher Scientific). Clones were digested with BamHI and KpnI (5′ region of *secA2)* or KpnI and HindIII (3′ end of *secA2)* and cloned into BamHI-HindIII digested pMV10-25 vector. The pMV10-25 SecA2 complement clone was electroporated into the electrocompetent Δ*secA2* strain and colonies selected on plates containing hygromycin. Colonies were screened by PCR to confirm the complementation of the *secA2* gene.

A list of all strains used in this study is provided in Table 1.

**Table 1:** List of strains used in this study

Strain name	Description	References
*Mycobacterium smegmatis*	*Mycobacterium smegmatis* mc^2^ 155 electroporation proficient WT strain	
*Mycobacterium smegmatis* VC	*Mycobacterium smegmatis* WT harboring episomal pMV10-25 plasmid (Hyg^+^)	This study
*Mycobacterium smematis* Rv1636 OE	*Mycobacterium smegmatis* WT harboring episomal pMV10-25-P_*rv1636*_*-rv1636* plasmid (Hyg^+^)	This study
*Δmsmeg_3811*	*M. smegmatis* strain deleted for the endogenous *msmeg_3811* gene (unmarked)	This study
*Mycobacterium tuberculosis*	*M. tuberculosis* H37Rv WT strain	
*Mtb::rv1636*	*M. tuberculosis* WT strain having an integrated copy of *rv1636* gene at the L5 *att* site as pMV306-P_*rv1636*_*-rv1636* (Kan^+^)	This study
*Mtb::rv1636*^*DM*^	*M. tuberculosis* WT strain having an integrated copy of *rv1636*^*DM*^ allele at the L5 *att* site as pMV306-P_*rv1636*_*-rv1636*^*DM*^ (Kan^+^)	This study
*Δrv1636::rv1636*	*M. tuberculosis* strain deleted for the endogenous *rv1636* gene, but harboring an additional copy at the L5 *att* site as pMV306-P_*rv1636*_*-rv1636* (Kan^+^)	This study
*ΔRD1*	*M. tuberculosis* H37Rv strain deleted for the *RD1* locus	(66, 67)
*ΔsecA2*	*M. tuberculosis* H37Rv strain deleted for the *secA2* gene	(30)
*Mtb::cfp10-nluc*	*M. tuberculosis* WT strain harboring integrated copy of pMV306-P_*cfp10*_*-cfp10-nluc* at the L5 *att* site (Kan^+^)	This study
*ΔsecA2::secA2*	*M. tuberculosis* H37Rv *ΔsecA2* strain complemented with the WT *secA2* gene	This study
*Mtb::rv1636-nluc*	*M. tuberculosis* WT strain harboring integrated copy of pMV306-P_*rv1636*_*-rv1636-nluc* at the L5 *att* site (Kan^+^)	This study
*Mtb::nluc*	*M. tuberculosis* WT strain harboring integrated copy of pMV306-P_*rv1636*_*-nluc* at the L5 *att* site (Kan^+^)	This study
*ΔRD1::cfp10-nluc*	*M. tuberculosis ΔRD1* strain harboring integrated copy of pMV306-P_*cfp10*_*-cfp10-nluc* at the L5 *att* site (Kan^+^)	This study
*ΔRD1::rv1636-nluc*	*M. tuberculosis ΔRD1* strain harboring integrated copy of pMV306-P_*rv1636*_*-rv1636-nluc* at the L5 *att* site (Kan^+^)	This study
*ΔRD1::nluc*	*M. tuberculosis ΔRD1* strain harboring integrated copy of pMV306-P_*rv1636*_*-nluc* at the L5 *att* site (Kan^+^)	This study
*ΔsecA2::cfp10-nluc*	*M. tuberculosis ΔsecA2* strain harboring integrated copy of pMV306-P_*cfp10*_*-cfp10-nluc* at the L5 *att* site (Kan^+^)	This study
*ΔsecA2::rv1636-nluc*	*M. tuberculosis ΔsecA2* strain harboring integrated copy of pMV306-P_*rv1636*_*-rv1636-nluc* at the L5 *att* site (Kan^+^)	This study
*ΔsecA2::nluc*	*M. tuberculosis ΔsecA2* strain harboring integrated copy of pMV306-P_*rv1636*_*-nluc* at the L5 *att* site (Kan^+^)	This study
*Mtb::dCas9*	*M. tuberculosis* WT strain harboring integrated copy of pRH2502 plasmid encoding *S. pyogenes* dCas9 (Kan^+^)	(39) and this study
*Mtb::dCas9/scrambled-sgRNA*	*Mtb::dCas9* strain harboring the pRH2521 plasmid (Kan^+^ Hyg^+^)	(39) and this study
*Mtb::dCas9/rv1636-sgRNA*	*Mtb::dCas9* strain harboring the pRH2521-rv1636+11 plasmid (Kan^+^ Hyg^+^)	This study

### Immunoblotting

Protein samples were electrophoresed on SDS-polyacrylamide gel of appropriate percentage and transferred to polyvinylidene fluoride membrane for immunoblotting. Blots were probed with the primary antibody in TBST (10 mM Tris–Cl (pH 7.5), 0.9% NaCl, and 0.1% Tween 20) containing 0.2% bovine serum albumin (TBT) overnight at 4 °C followed by horseradish peroxidase (HRP)-conjugated secondary anti-rabbit immunoglobulin G antibody (1:50,000) for 1 h at room temperature (RT). Bound antibody was detected using enhanced chemiluminescence with Immobilon Western HRP Substrate (Merck Millipore). A total of 20 to 25 μg of protein was loaded for whole cell lysates and sub-cellular fractions, whereas 25 ng was loaded for purified recombinant Rv1636. Antisera against Rv1636 was raised by immunizing a rabbit with purified, recombinant Rv1636. Anti-CRP (59) and anti-CFP-10 (BEI Resources, NIAID, National Institutes of Health, USA) antisera were used at recommended dilutions. Anti-CRP and anti-GyrB antibodies were described earlier and available in the laboratory (59, 60).

### Preparation of culture filtrate, cell envelope, and cytosolic fractions

To prepare culture filtrate from different strains of *M. tuberculosis* H37Rv cultures, cells were grown in 100 ml of 7H9 medium containing 0.2% glycerol in roller culture bottles for 4 weeks at 37 °C. Cells were harvested by centrifugation; culture supernatant was collected and filtered through a 0.2 μm syringe filter. Culture supernatant proteins were precipitated using 20% trichloroacetic acid at −20 °C overnight and centrifuged at 20,000*g* at 4 °C for 1 h. Proteins were washed twice with ice-cold acetone, and pellets were air-dried and resuspended in 150 mM Tris–Cl (pH 8) (61). Equal volumes of the resuspended protein for different strains were mixed with SDS loading dye, heated at 95 °C for 10 min, and loaded for analysis by SDS-PAGE, followed by Western blotting.

The cell pellets were washed with cold PBS and resuspended in lysis buffer containing 50 mM Tris–Cl (pH 8.2), 100 mM NaCl, 10 mM 2-mercaptoethanol (2-ME), 10% glycerol, and 1 mM PMSF. Cells were lysed by bead beating, and centrifuged at 1000*g* for 1 min, followed by harvesting of the supernatant and further centrifugation at 17,000*g* for 20 min at 4 °C. The supernatant fraction was used as the cytosol, while the pellet was resuspended in fresh lysis buffer and used as the cell envelope fraction. Protein samples were quantified by the Bradford method, and equal protein (20–25 μg) was loaded for SDS-PAGE followed by immunoblotting.

### Monitoring secretion of Rv1636 using a nanoluciferase assay

The nanoluciferase (Nluc) gene was obtained from the pNL1.1 plasmid (Promega Corporation). The nanoluciferase encoding gene was fused to the C terminus of CFP-10 or Rv1636 following a short linker sequence (GGSGGGGSGGGSSGG) described earlier for other fusion constructs (34). The *cfp10-nluc* and *rv1636-nluc* constructs were cloned under the respective endogenous *cfp-10* and *rv1636* promoters in the pMV306 vector. The *nluc* gene alone was cloned under the *rv1636* promoter as a control. Each construct was electroporated in WT, *ΔRD1,* and *ΔsecA2 M. tuberculosis* strains (Table 1). The strains were grown in 7H9 medium containing 0.2% glycerol, 0.05% Tween 80, and 10% OADC supplement with 20 μg/ml kanamycin till *A*_595nm_ reached ∼1 in 24-well plates. The cells were pelleted, washed twice with fresh medium, and finally resuspended in fresh 7H9 medium containing 0.2% glycerol, 0.05% Tween 80, and 20 μg/ml kanamycin such that the *A*_595nm_ was 1. The cultures were incubated at 37 °C harvested at different times, and culture supernatants were collected by centrifugation of the suspension at 12,000*g* for 5 min at RT.

For measuring Nluc activity, culture supernatants were assayed using 2.5 μM coelenterazine 400a as substrate. The assay buffer was essentially used as described by Hall *et al.* (34) and consisted of 50 mM Mes (pH 6), 1 mM EDTA, 0.5% NP-40, 150 mM KCl, 1 mM DTT, 35 mM thiourea. and 2.5 μM coelenterazine 400a. For the assay, 40 μl of culture supernatant was mixed with equal volume of 2× assay buffer containing coelenterazine 400a, incubated for 3 min at RT, and luminescence counts were measured in a Tecan Infinite (m200 PRO) plate reader.

### Cyclic AMP measurement

Cyclic AMP measurements were performed using radioimmunoassay as described previously (2, 12, 37, 62). Briefly, for separation of intrabacterial bound and free cAMP, cells were harvested as the cells started entering the stationary phase (*A*_595nm_ ∼2–3). The cell pellets were washed with cold PBS, resuspended in lysis buffer containing 50 mM Tris–Cl (pH 8.2), 100 mM NaCl, 10 mM 2-ME, 10% glycerol, and 1 mM PMSF, and lysed by bead beating. The lysates were centrifuged at 1000*g* for 1 min, followed by harvesting of the supernatant and further centrifugation at 17,000*g* for 10 min at 4 °C. Next, protein (400 μg) in the supernatant (cytosolic fraction) was subjected to centrifugation through a 3 kDa cutoff membrane filter (Amicon Ultra-0.5 3-kDa Ultracel, Millipore) at 4 °C. Cyclic AMP was measured from the eluate (“free” cAMP) and neat cytosolic fractions (“total” cAMP), while the “bound” cAMP fraction was estimated by subtracting the “free” cAMP from the “total” cytosolic cAMP (12, 37).

Culture supernatant was used to measure the total extracellular cAMP concentrations. Cyclic AMP was measured by radioimmunoassay following acidification and acetylation of all samples.

### Southern blotting

Genomic DNA of *M. tuberculosis* strains was prepared as described earlier (63). Southern blotting to confirm the genotypes was performed using the ECL Direct Nucleic Acid Labeling and Detection kit (GE HealthCare). Probe (650 bp) specific to the *rv1636* allele (Fig. S1*A*) was prepared by PCR using Rv1636 5′KO Fwd and Rv1636 promoter Rev primers and nonradioactively labeled with HRP according to the manufacturer’s protocol. Hybridization was carried out at 42 °C postcapillary transfer of KpnI digested *M. tuberculosis* genomic DNA to Hybond-N+ nylon membrane (GE HealthCare). The blot was developed using chemiluminescent substrates (Immobilon Western HRP Substrate, Merck Millipore). Sequences of all primers used in this study are shown in Table 2.

**Table 2:** List of primers used in this study

Primer name	5′ to 3′ sequence
Rv1636 5′KO Fwd	GATCAAGCTTACCGGTCGCCGGCGGCGGCCGCTCC
Rv1636 5′KO Rev	GATCGAATTCAATATTCGACGAGTCCGAACCGTCGG
Rv1636 3′KO Fwd	GATCGAATTCCCATGGAATGTGTCACGCCGGGCCAAG
Rv1636 3′KO Rev	ATGCGGATCCTCGAACGCGGCAAGGCGGCGC
Rv1636 KOscreen Fwd	GCAAATGAGGGTTGCGCCACG
Rv1636 KOscreen Rev	CGTGTACGCCGACTCCACCGTC
MSMEG_3811 KOscreen Fwd	GTTTGCCTGCTGTGCGTGCGG
MSMEG_3811 KOscreen Rev	CGTCTACGACGACCGCACGGTC
MSMEG_3811 5′KO Fwd	GATCAAGCTTTCGATCAGCCCGAACC
MSMEG_3811 5′KO Rev	ATCGAGAATTCCGACGAATCGGATCCG
MSMEG_3811 3′KO Fwd	ATCGAGAATTCGCGAACGTGGCACGCC
MSMEG_3811 3′KO Rev	GATCGGTACCAGCCAGGAACCGCAGC
Rv1636 RT Fwd	TCGTCGGCGCCCCGGTCGACGC
Rv1636 RT Rev	TTSGCSGGYACCGAKCCGAGCAG
Rv1636 promoter Rev	ATGCACCGGTTTACCCTCCCAGGTG
MSMEG_3811 promoter Rev	ATGCGGTACCCCATACCCTCCCACTG
Rv1636 ClaI Rev	ATGCATCGATCTAGGTGGTGTGCACG
MSMEG_3811 ClaI Rev	ATGCATCGATCTAGCTGGTGTGGAC
Rv1636 SCO Orientation Fwd	GCTTCGATTCCGGCCCACG
CFP10 Prom HindIII Fwd	ATGCAAGCTTACTGGTGAGCTCCCGTAATG
CFP10 HindIII Fwd	ATGCAAGCTTATGGCAGAGATGAAGACC
CFP10 fusion BamHI Rev	ATGCGGATCCACCACCGCTACCTCCGAAGCCCATTTGCGAGG
Mtb attB Fwd	GATGTCTGTCACCACGTACAGTCGC
Rv1636 fusion Rev	ATGCGGATCCACCACCGCTACCTCCGGTGGTGTGCACGATC
Nluc fusion Fwd	ATGCGGATCCTCGGGTGGTATGGTCTTCACACTC
Nluc Rev	ATGCTCTAGATTACGCCAGAATGCG
Rv1636 +11 CRISPRi bottom	AAACATAAGACCGTGGTGGTAGGA
Rv1636 +11 CRISPRi top	GGGATCCTACCACCACGGTCTTAT
Rv1636 G10T	AAGACCGTGGTGGTAACAACCGACGGTTCGGAC
Rv1636 G113A	CCTGCTGGTCGTCGCCAATGTCGGTCTGAGC
SecA2_first BamH1 fwd	CGCGGATCCGTGAACGTGCACGGTTGTCC
SecA2_first Kpn1 rvs	AAAGGTACCGACCAGCACAGGCTGCCCCC
SecA2_second Kpn1 fwd	ATCGGTACCCGCGACGTGGCCGAATCCGA
SecA2_second HindIII rvs	GGGAAGCTTTCAGCGGAACACCCCGGGCA

### Generation of *Δmsmeg_3811* and *Δrv1636* strains

The *msmeg_3811* gene was deleted in WT *M. smegmatis* strain following the protocol described by Parish and Stoker (38). For generation of the KO, 650 nucleotides upstream and downstream of *msmeg_3811* and 51 nucleotides from the start and before the stop codon of the *msmeg_3811* ORF, respectively, were cloned into the p2NIL vector to generate the p2NIL-*msmeg_3811*KO construct. The *Pac*I fragment from pGOAL19 containing three markers (β-galactosidase, hygromycin resistance, and sucrose susceptibility (*sacB*)) was cloned into p2NIL-*msmeg_3811*KO to generate plasmid p2NIL-*msmeg_3811*KO-*Pac*I. Subsequently, 5 to 10 μg of p2NIL-*msmeg_3811*KO-*Pac*I was electroporated into WT *M. smegmatis* electrocompetent cells. SCO colonies were selected using kanamycin, hygromycin, and X-gal markers. SCOs were allowed for the second crossover, and the positive colonies were selected using sucrose and X-gal. The DCO were further confirmed using genomic PCR and immunoblotting (Fig. 5*B*).

For deletion of the *rv1636* gene from WT *M. tuberculosis* H37Rv, a similar two-step selection strategy was used as that for *msmeg_3811* KO generation (38). A total of 650 nucleotides upstream and downstream of *rv1636* and 51 nucleotides from the start and before the end of the *rv1636* ORF were cloned into the p2NIL vector to generate the p2NIL-*rv1636*KO construct. Two merodiploid strains were generated by integrating either a WT copy of the *rv1636* gene or an *rv1636*^*DM*^ allele at the L5 *att* site of the *M. tuberculosis* genome, to generate *Mtb::rv1636* and *Mtb::rv1636*^*DM*^ strains, respectively. SCO colonies were confirmed by PCR and Southern blotting (Fig. S1*B*), and positive colonies were proceeded with for the second crossover. All DCO colonies appearing at the end of the process were screened by PCR using specific primers for the deletion of the endogenous *rv1636* gene (Fig. S1*A*).

### CRISPRi-mediated knockdown of *rv1636*

CRISPRi-based knockdown of *rv1636* in *M. tuberculosis* H37Rv was performed using the strategy described initially by Singh *et al.* (39). First, the codon-optimized *Streptococcus pyogenes* dCas9 expressing integrative plasmid pRH2502 was introduced into WT *M. tuberculosis* H37Rv to generate the strain *Mtb::dCas9*. A 20 nt-long *rv1636* targeting spacer sgRNA sequence (+11 to +30 positions within the *rv1636* ORF) was cloned within the two BbsI sites in the pRH2521 plasmid to generate pRH2521-Rv1636 + 11 construct. Either the episomal pRH2521 (scrambled nontargeting control) or pRH2521-Rv1636 + 11 plasmids were transformed into *Mtb::dCas9* strain to generate *Mtb::dCas9/scrambled-sgRNA* and *Mtb::dCas9/rv1636-sgRNA* strains, respectively. Cultures were grown in Middlebrook 7H9 broth, supplemented with 0.2% glycerol, 10% OADC, 0.05% Tween, 50 μg/ml hygromycin, and 25 μg/ml kanamycin, under static conditions at 37 °C till they reached an absorbance of 0.2. Then 5 μl of cultures were spotted on 7H10 agar media containing 10% OADC enrichment and 0.2% glycerol or plates containing ATC (100 ng/ml) and incubated for 6 to 7 days.

Suspension cultures were grown to an absorbance of 0.4, harvested 2 and 4 days after treatment with ATC, with ATC replenished every 48 h. Lysates were prepared and subjected to Western blot analysis with an antibody to Rv1636 as described above.

### Biolayer interferometry

Protein binding to cAMP was monitored using biolayer interferometry (BLI) on the Octet RED96 system (Pall ForteBio LLC). Recombinant Rv1636^DM^ (Rv1636^G10T G113A^) was generated by site-directed mutagenesis, taking pPRO-Rv1636 plasmid as the template (12, 64). Recombinant Rv1636 and Rv1636^DM^ proteins were expressed and purified from *Escherichia coli* SP850 *cyc*^*−*^ strain (65) as described previously (12). For BLI, 1 mM 8-(6-aminohexylamino) cAMP (8-AHA-cAMP; Biolog) prepared in 100 mM Hepes (pH 8) was used for conjugation (1200 s) to AR2G tips (amine-reactive) after activation (720 s) with a mixture of 20 mM 1-ethyl-3-(3-dimethylaminopropyl)carbodiimide and 10 mM N-hydroxysuccinimide in a final reaction volume of 200 μl. The reaction was quenched by adding 1 M ethanolamine–HCl (pH 8.5). Reference tips were activated and deactivated without the addition of 8-AHA-cAMP. All interactions were studied in buffer containing 10 mM Hepes (pH 7.5), 100 mM NaCl, 10 mM 2-ME, 10% glycerol, 0.5% bovine serum albumin, and 0.05% Triton X-100. All proteins were used at a concentration of 4 μM. The association and dissociation were monitored for 10 min at 25 °C. After the interaction, the tips were regenerated with 0.05% SDS prepared in the assay buffer. Binding kinetics were analyzed using Octet Data Analysis software, version 8.2 (Pall ForteBio LLC).

### Statistical analyses

All graphs were plotted, and statistical analyses were performed using GraphPad Prism 10 (https://www.graphpad.com).

## Data availability

All data are contained within the manuscript.

## Supporting information

This article contains supporting information.

## Conflict of interest

The authors declare that they have no conflicts of interest with the contents of this article.
