# Supplementary material for: Cyclic AMP binding to a universal stress protein in Mycobacterium tuberculosis is essential for viability
Source: J Biol Chem. 2024 Apr 16;300(5):107287. doi: 10.1016/j.jbc.2024.107287 (PMC11107214; doi:10.1016/j.jbc.2024.107287)
Supplement: Supporting Figure S1 [file mmc1.pdf]

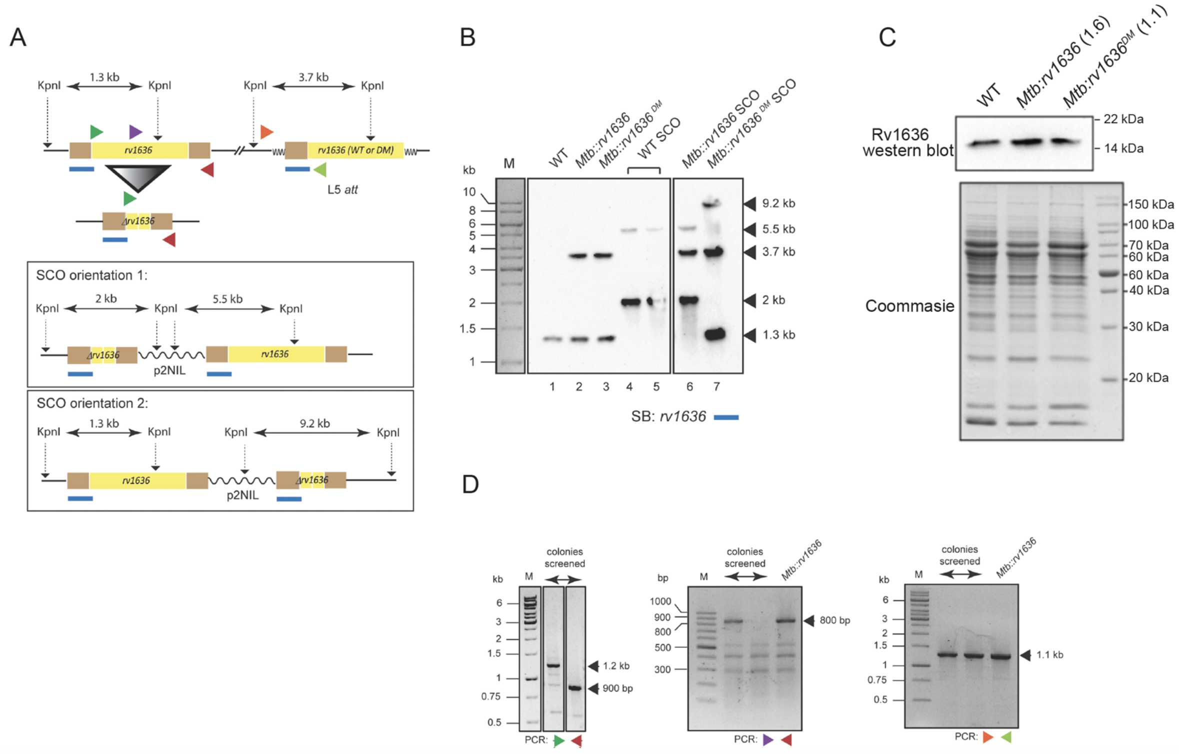

Supplemental Figure 1. The *rv1636* gene is essential in *M. tuberculosis,* and essentiality is dependent on its cAMP-binding ability. (A) Schematic representation of the wildtype and *Δrv1636* alleles, the yellow bar representing the gene ORF, while the flanking genomic regions are depicted in brown. At the L5 *att* site, the additional *rv1636* allele containing ~650 nt upstream region (brown) along with the ORF (yellow) represents the DNA fragment used to construct the merodiploid strains. The blue line indicates the region used to probe the *rv1636* allele in Southern blot. The PCR primer pairs used in screening are depicted as color-coded arrowheads. Image not to scale. Schematic of KpnI digested fragments shown in Supplemental Fig 1B are shown for the two possible orientations of single cross-over strains. (B) Southern blot to confirm the genotypes of the following *M. tuberculosis* strains – wildtype (WT), merodiploid having an additional copy of either the wildtype (*Mtb::rv1636*) or double mutant *rv1636* (*Mtb::rv1636*^{DM}) alleles, wildtype single crossovers (WT SCO), and single crossovers in the merodiploid backgrounds, respectively. The number of alleles in the respective strains is detected by the number of bands at their corresponding sizes. Genomic DNA was digested with *Kpn*I prior to transfer and blotting. (C) Immunoblot showing the expression levels of Rv1636 in the merodiploid strains *Mtb::rv1636* and *Mtb::rv1636*^{DM}, respectively, compared to wildtype *M. tuberculosis*. Numbers next to the label represent the relative densitometric amounts across two experiments. The bottom panel shows a Coommasie stained gel following SDS-PAGE of lysates used for immunoblotting. (D) Left panel: PCR confirming deletion at the endogenous locus after DCO. WT allele gives a 1.2 kb amplicon while a deletion allele gives a 900 bp product. Middle panel: PCR to confirm DCO colonies from *Mtb::rv1636* where only the wildtype allele at the endogenous locus will generate an 800 bp product. The absence of this product confirms the deletion of the endogenous *rv1636* gene (third lane from the left). Right panel: PCR to confirm the presence of the *rv1636* allele at the L5 *att* site in the DCO colonies shown in Fig 6D, left panel.
